# Viewing alcohol warning advertising reduces urges to drink in young adults: an online experiment

**DOI:** 10.1186/s12889-016-3192-9

**Published:** 2016-07-08

**Authors:** Kaidy Stautz, Theresa M Marteau

**Affiliations:** Behaviour and Health Research Unit, Institute of Public Health, University of Cambridge School of Clinical Medicine, Box 113 Cambridge Biomedical Campus, Cambridge, CB2 0SR UK

**Keywords:** Alcohol, Alcohol advertising, Alcohol marketing, Alcohol warnings, Craving

## Abstract

**Background:**

Tobacco counter-advertising is effective at promoting smoking cessation. Few studies have evaluated the impact of alcohol warning advertising on alcohol consumption and possible mechanisms of effect. This pilot study aimed to assess whether alcohol warning advertising is effective in reducing urges to drink alcohol, if emotional responses to advertising explain any such effect or perceived effectiveness, and whether effects differ among heavier drinkers.

**Methods:**

One hundred fifty-two young adult (aged 18–25) alcohol users completed an online experiment in which they were randomly assigned to view one of three sets of six advertisements: (i) alcohol warning; (ii) alcohol promoting; or (iii) advertisements for non-alcohol products. Urges to drink alcohol were self-reported post-exposure. Affective responses (pleasure and arousal) to each advertisement and perceived effectiveness of each advertisement were recorded. Typical level of alcohol consumption was measured as a potential effect modifier.

**Results:**

Participants exposed to alcohol warning advertisements reported significantly lower urges to drink alcohol than those who viewed either alcohol promoting or non-alcohol advertisements. This effect was fully mediated by negative affective responses (displeasure) to the alcohol warning advertisements. Perceived effectiveness of alcohol warning advertisements was associated with high arousal responses. Impact of the advertisements was unaffected by typical level of alcohol consumption, although the study was not powered to detect anything other than large effects.

**Conclusions:**

In line with findings from the tobacco literature, alcohol warning advertisements that elicit negative affect reduce urges to drink alcohol. Their impact upon actual consumption awaits investigation.

**Electronic supplementary material:**

The online version of this article (doi:10.1186/s12889-016-3192-9) contains supplementary material, which is available to authorized users.

## Background

It is estimated that 5.9 % of global deaths and 5.1 % of the global burden of disease are attributable to alcohol consumption [1]. In England, although young adults are less likely than other age groups to report drinking any alcohol at all, they are the age group most likely to report very excessive alcohol consumption (over 8 units) on at least one day in the previous week [2]. Among females, those in the 16–24 age group have the highest prevalence of hazardous and harmful drinking and of alcohol dependence [3], whilst among young adult males there was a 57 % increase in alcohol-related hospital admissions from 2002 to 2010 [4].

Young adults are frequently the target of alcohol marketing [5]. One policy option to reduce harmful alcohol consumption in this age group is to restrict the marketing of alcoholic products. However, despite consistent evidence that exposure to alcohol advertising has a dose–response association with earlier initiation of alcohol use and increased consumption in young people [6, 7], there is a lack of high quality evidence to support the implementation of alcohol marketing bans or restrictions to reduce alcohol consumption [8]. This is one reason that such strategies have not found favour with policymakers [9].

An alternative approach to restricting alcohol marketing is to counter alcohol advertising’s messages about the positive effects of alcohol consumption with public health messages presented as advertisements that warn about the negative consequences of alcohol use. The use of counter-advertising has been well studied in relation to tobacco, and there is a broad evidence base detailing strategies to maximise effectiveness [10, 11]. There is very little comparable evidence for alcohol counter-advertising. Whilst evidence exists suggesting that advertisement campaigns to reduce alcohol impaired driving can be effective when professionally executed [12, 13], there is very little information available as to whether campaigns focusing on more general negative consequences of alcohol use have any effect in reducing alcohol consumption or changing the psychological predictors of consumption, such as attitudes towards alcohol, expectancies of use, and desire or craving for alcohol.

In addition to assessing *if* alcohol warning advertisements are effective, it is necessary to understand *how* they are effective so that campaigns can be designed to have maximum impact. Findings from the tobacco counter-advertising literature indicate that advertisements evoking strong negative emotions, such as fear and disgust elicited by graphic imagery of the damaging health effects of smoking or anger elicited by highlighting disingenuous practices of the tobacco industry, are more likely to promote smoking cessation, reduce the intention to smoke, and to have high perceived effectiveness (i.e. the perception that advertisements will be effective in convincing smokers to reduce or quit) [10, 14, 15]. There is also indication that emotionally evocative advertisements have the potential to reduce socioeconomic inequalities in smoking [14]. As yet, it is not known whether alcohol warning advertising that elicits strong negative emotional responses is similarly potent in influencing alcohol-related cognition or behaviour.

A further consideration in developing alcohol warning messages is the possibility for counterintuitive ‘boomerang’ effects whereby exposure to the message increases the likelihood of the behaviour being warned against. There is some evidence of a boomerang effect in response to alcohol warning advertisements among heavier drinkers. In a recent study, heavier drinkers who viewed alcohol warning advertisements showed a reduction in negative implicit attitudes towards alcohol after viewing, compared to lighter drinkers [16]. This result may be explained by incentive sensitisation theory [17], which suggests that alcohol cues such as those present in alcohol warning advertising (i.e. words and images related to alcohol) might stimulate alcohol craving among heavy drinkers. In the current study, heaviness of drinking will be tested as a moderator of any effect of condition.

### Aims and Hypotheses

The primary aim of this pilot study is to assess whether exposure to alcohol warning advertising is effective in reducing the urge to drink alcohol, and whether affective responses to advertising help to explain any such effect. We predict that participants exposed to alcohol warning advertisements will report fewer urges to drink alcohol compared to those exposed to alcohol promoting or non-alcohol advertisements (*H1*), and that, if present, this effect will be mediated by affective responses (low pleasure and high arousal) to advertisements (*H2*). The second aim is to assess whether any effects of alcohol-related advertising on urges to drink alcohol are stronger amongst heavier drinkers. We predict that heavier drinkers exposed to alcohol warning advertisements will report higher urges to drink compared to those exposed to non-alcohol advertisements (*H3*). The third aim is to assess whether emotional responses to advertisements are associated with their perceived effectiveness. We predict that affective responses to alcohol warning advertisements (low pleasure and high arousal) will be associated with higher perceived effectiveness (*H4*). The fourth aim is to identify appropriate advertisements for use in a laboratory-based study on the impact of alcohol-related advertising on alcohol consumption in young adults.

## Method

### Participants

Participants comprised 152 young adults (50 % female, 49.3 % male, 1 did not report gender). Inclusion criteria were that participants were aged between 18 and 25 and were current alcohol users, defined as drinking at least one alcoholic beverage during a typical week. The mean age was 21.47 (SD = 1.31). The majority of the sample reported their ethnicity as ‘White British’ (65.1 %) or ‘Any other White background’ (17.8 %). Participants were recruited via a UK based research agency, *YouthSight* (youthsight.com), that sent a request for participation to members of their existing panel of over 135,000 16–30 year olds residing in the UK. Participants who responded to this request and met our inclusion criteria, assessed using an online pre-screening questionnaire, were given a web link to complete the study using the Qualtrics survey platform. Participants were paid £6 for participation. This incentive was delivered via the agency to those who completed the study.

As the current study used a pilot sample to test exploratory research questions, a formal sample size calculation was not conducted and the sample size was based on practical constraints. The study had the power to detect medium to large effects (*f* = ~0.26) with 80 % power using an alpha value of 0.05.

### Design

This study used a between-participants experimental design, whereby participants were randomly assigned to view one of three sets of advertisements: (i) alcohol warning, (ii) alcohol promoting, or (iii) non-alcohol advertisements, before completing a post-exposure outcome measure. Randomization was conducted by the Qualtrics Randomizer function, which was set to assign an equal number of participants to each condition. Participants were blind to conditions other than their own until participation was complete, at which point they were debriefed about the study aims.

### Procedure

The study was completed entirely online. Participants gave informed consent and then completed a battery of questionnaires. Following the questionnaires, participants were presented with a random selection of six of 15 condition-specific advertisements. Following each advertisement, participants reported their current pleasure and arousal, and the degree to which they perceived the advertisement to be effective. After rating six advertisements, participants reported their urges to drink alcohol. At the end of the study participants were shown a debrief page that included a link to online information about alcohol and health [18].

### Stimuli

A total of 45 advertisements (15 per condition) were selected for the study. Alcohol warning advertisements were identified by searching YouTube with the terms ‘alcohol warning’, ‘anti-alcohol’, and ‘alcohol AND health’. Criteria for inclusion were that advertisements were professionally produced, appeared to be relevant to young adults, and highlighted short-or long-term negative consequences of alcohol consumption. Selected advertisements were produced between 2006 and 2015 in the United Kingdom (7 advertisements), Australia (4), New Zealand (1), the Republic of Ireland (1), Sweden (1), and Iceland (1). All advertisements were presented in English language. Table 1 describes the advertisements used and the message content (i.e. type of negative consequence emphasised) and presentation style of each advertisement. Categories of message content were: injury; short-term health effects (e.g. vomiting, loss of consciousness); long-term health effects (e.g. cancer); social consequences (e.g. embarrassment, offending friends); harm to others (e.g. accidental physical harm, abuse, use of public services); and criminal behaviour (e.g. violence, being arrested). Categories of presentation style were: graphic (using shocking aversive images such as vomiting, injuries); depiction (acted scenes of intoxication); testimonial (real or acted description of events); and animated text (text corresponding with voiceover). Categories were adapted from a study of obesity prevention advertisements [19], and were coded by the first author.

**Table 1:** Alcohol warning advertisements presented in the study, ranked by mean perceived effectiveness

Title	Country	Duration (seconds)	Message content	Presentation style	Perceived effectiveness
Know your limits (Male)	UK	39	Injury, social consequences	Graphic, depiction	6.68
It’s how we’re drinking	New Zealand	45	Harm to others	Graphic, depiction	6.59
Rethink drink	Australia	38	Harm to others	Depiction	6.47
Another night wasted	UK	40	Short-term health effects, social consequences	Graphic, depiction	6.44
Don’t turn a night out into a nightmare	Australia	90	Injury	Graphic, testimonial	6.09
What you can’t see	Australia	30	Long-term health effects	Depiction	6.00
Tumour	UK	40	Long-term health effects	Graphic	5.90
Smooth	Sweden	60	Short-term effects, harm to others, dependence	Depiction	5.78
Who is in control	UK	69	Short-term health effects, social consequences	Graphic, depiction	5.57
Had enough	Rep. of Ireland	42	Harm to others	Depiction	5.52
Superhero	UK	43	Injury	Graphic, depiction	5.47
I See	Australia	45	Short- and long-term health effects, social consequences	Graphic, testimonial	5.47
Know your limits (Female)	UK	40	Injury, social consequences	Graphic, depiction	5.06
Don’t drink like a pig	Iceland	52	Harm to others, criminal behaviour	Depiction	4.60
You wouldn’t sober	UK	40	Harm to others	Animated text	4.41

Alcohol promoting and non-alcohol advertisements were selected based on data from a 2014 survey of the UK’s most popular brands amongst 18–24 year olds [20]. Advertisements were identified on official YouTube accounts of popular brands in May 2015. Selected advertisements were all uploaded to YouTube within the previous year. Non-alcohol advertisements were for electronic products, clothing stores, and online services. No advertisements for food or drink products were included in this condition.

Duration of individual advertisements ranged from 20 to 107 seconds. Mean duration of advertisements in each condition was: alcohol warning = 47.53, alcohol promoting = 54.27 seconds, non-alcohol = 55.07.

## Measures

### Outcome measure

#### Urge to consume alcohol

The Alcohol Urge Questionnaire [21] was used to assess current desire to consume alcohol, an index of alcohol craving. This measure contains eight items, each with a seven point response scale ranging from “*1*–*Strongly Disagree*” to “*7*–*Strongly Agree*” with a neutral option. A sample item is “*Nothing would be better than having a drink right now*”. Scores are continuous and range from 8 (low urge to drink) to 56. The AUQ has been shown to have concurrent validity, construct validity, and good test-retest reliability [21]. The measure showed high internal consistency in the current sample, with a Cronbach’s alpha of .87.

### Proposed mediators

#### Affective responses to advertisements

Pleasure (versus displeasure) and arousal (versus tiredness) were assessed immediately after each advertisement. These two aspects of momentary core affect are purported to be orthogonal dimensions, such that affective states can be high in pleasure and arousal (e.g. excitement, joy), low in pleasure and high in arousal (e.g. rage), or any combination of the two [22]. Pleasure was assessed with the item “*How pleasant did this advertisement make you feel*?”, whilst arousal was assessed with “*How alert did this advertisement make you feel*?” Responses were given on two 11 point visual analogue scales, anchored with “*0*–*Very unpleasant and negative*” to “*10*–*Very pleasant and positive*” for pleasure; and “*0*–*Inactive and tired*” to “*10*–*Alert and energetic*” for arousal. Items were adapted from the Affect Grid [23]. This brief measure is recommended for contexts where participants are asked to make multiple affective judgements over a short space of time [23]. The pleasure scale has been shown to be a valid marker of momentary affect along the pleasure-displeasure dimension [24]. Affective responses to the six viewed advertisements were summed and averaged to provide two continuous summary scores of momentary pleasure and arousal. Cronbach’s alpha values indicated high consistency in participants’ pleasure responses (.87), though weaker consistency in arousal responses (.67).

### Covariates

#### Alcohol use

The Alcohol Use Disorders Identification Test (AUDIT [25]) was used to assess heaviness of drinking. Scores range from 0 to 40, with higher scores reflecting more hazardous and harmful alcohol use. The AUDIT has been shown to have good construct validity for assessing alcohol consumption and adverse consequences of drinking, and a high degree of internal consistency and test-retest reliability [26]. The AUDIT had good internal consistency in this sample, with a Cronbach’s alpha value of .76. Scores were treated as continuous.

#### Typical media use

Two items were included to control for participants’ typical exposure to video advertising. Typical hours per day of television usage was assessed with the item: “*On average*, *how many hours per day do you watch television*”. Typical hours per day of recreational internet use was assessed with the item “*On average*, *how many hours per day do you use the internet for non*-*work purposes*?” Scores for each variable were from 0–20 and treated as continuous.

### Additional measures

#### Perceived effectiveness

Participants exposed to alcohol warning advertisements responded to the item “*How effective do you think this advertisement is in encouraging people to reduce their alcohol consumption*?” by placing a mark on an 11 point visual analogue scale anchored with “*0*–*Not at all effective*” to “*10*–*Extremely effective*”. The format of this question was adapted from a previous study [27]. Responses to the six viewed advertisements were summed and averaged for a total continuous score.

### Demographics

Age, gender, ethnicity, subjective social class were assessed by self-report.

### Understanding of ‘Drink Responsibly’

Participants were asked “*What do you think is meant by the term* ‘*Drink Responsibly*’?” with an open-ended response format. This item served to collect qualitative information about young adults’ understanding of this term, which has been shown to lack consensus in previous research [28], and also to assess participant engagement with the study. Participants who provided no response or a nonsensical answer to the item were screened out, as were those who answered over ‘20’ for typical hours of television or internet used each day.

### Data analysis

Data were analysed using IBM SPSS Statistics Version 22. Heaviness of drinking, typical television use, and typical internet use scores showed modest positive skew (skewness values all slightly over 1.0) and were square root transformed prior to analysis to meet the assumption of normality. Data met assumptions of independence and homoscedasticity, assessed by visual inspection of residual plots and by Levene’s test. The test for linearity function within SPSS was used to confirm that covariates and potential mediators showed linear relationships with the dependent variable (departures from linearity all *p* > .05). The effectiveness of randomisation was determined using one way ANOVAs and independent *t* tests to assess for group differences in demographic and alcohol-related characteristics. Differences in mean urges to drink scores between conditions and the interaction between condition and heaviness of drinking were tested using ANCOVAs, with condition and gender (coded as 0 = female, 1 = male) as factors and heaviness of drinking, television use, and internet use included as covariates. Significant effects of condition were probed using planned pairwise comparisons. To assess indirect effects of experimental condition on urges to drink via affective responses to advertisements, multiple mediation analysis was conducted with the SPSS PROCESS macro, model 4 [29]. The PROCESS macro employs an ordinary least squares regression-based path analytic framework to estimate direct and indirect effects in multiple mediator models. Bias corrected bootstrapping with 5000 samples was used to ascertain 95 % confidence intervals. Heaviness of drinking, typical television use, and typical internet use were entered as covariates in the model. To assess whether pleasure and arousal responses to alcohol warning advertisements were associated with their perceived effectiveness, partial correlations between these variables were conducted, controlling for heaviness of drinking.

## Results

### Sample characteristics

‘Catch’ questions identified and screened out 32 participants who appeared not to be fully engaging with the study (leaving a study sample of 152). Table 2 presents participant characteristics. There were no differences between conditions in age, socio-economic status, heaviness of drinking, or television use. Internet use varied by group, with those in the alcohol promoting advertisements condition reporting using the internet less than those in both the alcohol warning (*t* (100) = −2.32, *p* = .02) and non-alcohol (*t* (100) = −2.99, *p* = .004) conditions. Internet use was included as a control variable in all further analyses. Across the entire sample, the Pearson correlation between pleasure and arousal responses to advertisements was *r* = .28 (*p* < .001), indicating that these were related yet separate constructs.

**Table 2:** Participant characteristics in total and by condition

	Total	Condition		
		Alcohol warning advertisements	Alcohol promoting advertisements	Non-alcohol advertisements
*N*	152	50	52	50
Gender				
* Male*	75	26	22	27
* Female*	76	24	29	23
* Missing*	1	0	1	0
Age	M = 21.47 (SD = 1.31)	21.52 (1.33)	21.38 (1.37)	21.52 (1.23)
Ethnicity				
* White British*	99	35	35	29
* White Irish*	1	0	1	0
* Any other White background*	27	5	11	11
* Mixed White and Black African*	3	2	1	0
* Mixed White and Asian*	1	1	0	0
* Any other mixed background*	1	0	0	1
* Indian*	4	1	1	2
* Any other Asian background*	2	1	0	1
* Caribbean*	2	1	0	1
* Chinese*	6	1	2	3
* Prefer not to say*	1	1	0	0
* Missing*	5	2	1	2
Subjective social class				
* Working class*	34	12	12	10
* Lower middle*	35	7	16	12
* Middle class*	65	23	20	22
* Upper middle*	18	8	4	6
* Upper class*	0	0	0	0
Heaviness of drinking (AUDIT total score)	M = 8.80 (SD = 4.68)	9.92 (5.55)	8.35 (3.91)	8.14 (4.32)
Typical daily television use (hours)	M = 1.93 (SD = 1.92)	2.12 (2.33)	1.88 (1.40)	1.80 (1.97)
Typical daily internet use (hours)	M = 4.95 (SD = 2.82)	5.10 (2.56)	4.06 (1.96)	5.74 (3.53)

### Experimental effects

Table 3 presents mean scores on outcome measures across conditions. There was a significant difference in mean urges to drink scores between conditions, *F* (2, 142) = 7.10, *p* = .001. Pairwise comparisons of adjusted means indicated that participants in the alcohol warning condition reported fewer urges to drink alcohol than those in both the alcohol promoting (mean difference = −5.54, *p* < .001) and the non-alcohol (mean difference = −3.80, *p* = .01) conditions. The size of the effect of viewing alcohol warning advertisements, compared to non-alcohol advertisements, on urges to drink was *d* = −0.37. Participants’ urges to drink in the alcohol promoting and non-alcohol conditions did not differ (*p* = .26). There was a main effect of heaviness of drinking on urges to drink *F* (1, 142) = 19.40, *p* < .001. There was no evidence for an interaction between experimental condition and heaviness of drinking *F* (2, 141) = 0.32, *p* = .73. An additional analysis that replaced the transformed scores of three covariates in the model with untransformed scores indicated no notable differences in results.

**Table 3:** Mean (SD) scores on urges to drink alcohol, affective responses to advertisements, and perceived effectiveness

	Total	Alcohol warning advertisements	Alcohol promoting advertisements	Non-alcohol advertisements
Urges to drink alcohol	17.24 (8.12)	14.92 (7.27)	19.23 (9.59)	17.48 (6.68)
Affective responses				
* Pleasure*	5.04 (1.94)	3.16 (1.70)	5.83 (1.03)	6.10 (1.47)
* Arousal*	5.30 (1.22)	5.26 (1.18)	5.28 (1.11)	5.37 (1.39)
Perceived effectiveness	5.47 (1.47)	5.78 (1.45)	5.23 (1.57)	5.41 (1.35)

**Fig. 1:**
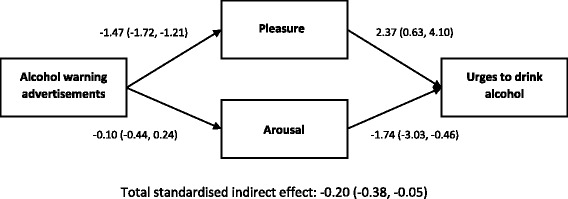
Multiple mediation model showing effect of viewing alcohol warning advertisements (compared to viewing alcohol promoting or non-alcohol advertisements) on urges to drink alcohol via mean pleasure and arousal in response to advertisements. Values are standardised regression coefficients with 95 % confidence intervals in parentheses. Heaviness of drinking, television use, and internet use were included in the model as covariates

An additional ANCOVA with mean pleasure and arousal scores included as covariates revealed effects of these variables on urges to drink (pleasure: *F* (1, 140) = 6.76, *p* = .01; arousal: *F* (1, 140) = 7.95, *p* = .005). The difference in mean urges to drink between conditions was no longer significant with pleasure and arousal included, *F* (2, 140) = 1.15, *p* = .32, suggesting that they mediate the impact of the intervention on urges to drink. To further probe this, we tested the indirect effect of condition (alcohol warning advertisements versus alcohol promoting and non-alcohol advertisements combined) on urges to drink via pleasure and arousal (Fig. 1). The *R*^*2*^ of the total effect model was .17 (*F* (4, 147) = 7.67, *p* < .001). There was an indirect effect of advertisement condition on urges to drink via low pleasure scores, β = −0.21, SE = 0.08, 95 % confidence interval = −0.38,–0.05. There was no indirect effect via arousal, β = 0.01, SE = 0.02, 95 % confidence interval = −0.02, 0.06.

### Predicting perceived effectiveness

The advertisement ‘*Know Your Limits* (*Male*)’, produced by the UK National Health Service and the Home Office, was the highest rated alcohol warning advertisement for perceived effectiveness with a mean score of 6.68 (Table 2). The lowest rated was ‘*You Wouldn*’*t Sober*’, produced by the alcohol industry funded body Drinkaware, which received a mean score of 4.41.

As our UK based sample was likely to be familiar with the alcohol warning advertisements from the UK and unfamiliar with those from other countries, we tested for any differences in mean perceived effectiveness scores between the UK based (*n* = 7) and the non-UK based (*n* = 8) advertisements and found no significant difference, M_UK_ = 5.65, SD = .64; M_Non-UK_ = 5.82, SD = .64; *t* (13) = 0.46, *p* = .65. There were also no differences in mean pleasure and arousal responses.

Partial correlation analysis indicated that the perceived effectiveness of alcohol warning advertisements was associated with high arousal responses to these advertisements (*r* = .50, *p* < .001). There was a non-significant negative association between perceived effectiveness and pleasure responses (*r* = −.24, *p* = .10).

### Understanding of ‘Drink Responsibly’

Participants’ understanding of the term ‘Drink Responsibly’ was assessed using a content analysis of open-ended responses (Additional file 1: Supplementary material S1). Ten themes were identified. The most popular themes expressed in this sample were: (i) to restrict levels of consumption, particularly in accordance with one’s personal limits (46.7 % of sample); (ii) to maintain control over behaviour and judgement (20.4 %); and (iii) to avoid health and safety risks to self and others (17.8 %).

## Discussion

This pilot study investigated the immediate impact of viewing alcohol warning, alcohol promoting, and non-alcohol advertisements on urges to drink alcohol. Our primary hypothesis was supported. Participants who viewed alcohol warning advertisements reported fewer urges to drink than those who viewed either alcohol promoting or non-alcohol advertisements. Our second hypothesis was partially supported: the effect of alcohol warning advertisements on reduced urges to drink alcohol was fully mediated by displeasure, although not by high arousal, felt in response to the advertisements. Our third hypothesis, that effects would differ among heavier drinkers, was not supported. In partial support of the fourth hypothesis, high arousal, but not pleasure, was associated with higher perceived effectiveness of alcohol warning advertisements.

These findings are in line with the literature on tobacco counter-advertising, which indicates that advertisements evoking negative emotion are particularly effective in promoting smoking cessation. Similarly, it has been shown that viewing aversive health-related images related to obesity alongside images of snack foods can make implicit attitudes towards these foods more negative and choice of such foods less likely [30, 31]. Taken in concert, these findings support the idea that negative imagery can influence health-related cognition and behaviour, perhaps via a priming process whereby viewing images of ill health activates motivation for good health. An understanding of how affective processes can be targeted to influence decision making and behaviour change may be beneficial in designing health communications.

Participants who viewed alcohol promoting advertising did not report greater urges to drink than those who viewed non-alcohol advertising. This may indicate that alcohol promoting advertising does not encourage alcohol use by stimulating alcohol craving. However, inducing or increasing alcohol craving using only alcohol-related imagery is known to be difficult in settings where no immediate opportunity for alcohol consumption is provided [32]. In addition, our index of alcohol craving – self-reported urges to drink alcohol – involved explicit, deliberative questions. Previous studies have shown that explicit alcohol attitudes are not influenced by exposure to alcohol advertising, but that implicit measures are [16, 33]. Future studies in this area should focus on implicit measurement of alcohol-related cognition.

The current findings should be considered alongside evidence that exposure to alcohol cues can produce negative affect and craving in alcohol dependent individuals [34]. We did not find evidence for a ‘boomerang’ effect of exposure to alcohol warning advertising in the total sample or in heavier drinkers specifically in this study. However, around half of participants were non-hazardous drinkers, and the study had limited power to detect such a moderation effect. It is not clear from these data whether the alcohol cues present in alcohol warning advertising could have adverse effects among dependent drinkers. More research is needed to clarify whether inducing certain negative emotional states through alcohol warning advertising may have iatrogenic effects in certain populations, or whether the pairing of alcohol cues with negative emotional imagery may even be an effective way of disrupting conditioned alcohol use patterns in dependent drinkers.

There was a positive association between average levels of arousal felt in response to the selection of alcohol warning advertisements viewed and the perceived effectiveness of these advertisements. This finding complements recent evidence on neural responses to graphic cigarette warning labels, which show that images evoking stronger affective responses also evoke patterns of brain activation related to hazard perception and emotional memory [35]. Emotionally salient, arousing images appear to facilitate transmission of the health message, memory of its content, and, as shown in this study, thoughts that the message will be effective. However, high arousal did not predict changes in urges to drink. This may be an important consideration when designing alcohol warning advertisements. Although people *perceive* messages containing shocking, arousing imagery to be influential, messages that induce negative affect (with or without high arousal) may be more likely to influence alcohol-related cognition and behaviour. Negative affect has been shown to impact upon information processing and motivation, reducing reliance on pre-existing knowledge and increasing the expected value of future achievements [36]. Motivations for future good health may also be stimulated by negative affect in response to health warnings.

### Implications

Producing alcohol warning advertising may be a valuable addition to policies aiming to reduce harmful alcohol consumption in young adults. The observation of reduced urges to drink immediately following exposure suggests that such advertisements could have an impact if presented at times and locations where urges to consume alcohol are highest, for instance on Friday and Saturday evenings [37] in or near to drinking establishments. This needs to be evaluated.

In the United Kingdom, the reliance on self-regulation of alcohol marketing by the alcohol industry has meant that few alcohol warning messages have been independently produced in recent years. Instead, responsible drinking messages, typically embedded within alcohol promoting advertising, have become common. Recent research suggests that responsible drinking messages have no clear definition, are predominantly used to promote alcohol products, and may encourage increased alcohol consumption [38, 39]. Indeed, many participants in the current study believed the term ‘drink responsibly’ meant to drink, but within unspecified personal limits. In contrast, the current findings suggest that alcohol warning messages, not produced by the alcohol industry, can be effective in reducing cravings to drink alcohol. The alcohol warning advertisements used highlighted various negative consequences that can results from excessive alcohol use, rather than focusing on responsible drinking messages. Future research might consider directly comparing the effectiveness of these two types of messages on alcohol-related cognition and behaviour. Most pertinently, evidence is needed on whether the type of messages used in the current study are effective in reducing alcohol consumption.

#### Strengths and limitations

This is the first study, to our knowledge, to examine the effects of alcohol warning advertising on urges to drink alcohol, a marker of problematic alcohol use [40]. The focus on affective processes is novel and aligns with current theories of health decision making that emphasise automatic processes [41]. There were, however, design limitations. As the study was conducted online participant understanding of the questions and tasks could not be monitored, although ‘catch’ questions were employed to screen out participants not attending fully to the study. Affective responses to advertisements were measured by self-report. Whilst such methods show some convergence with objective physiological measures [42], self-reports could be complemented by objective measures in future studies. All participants saw the same number of advertisements so we were unable to examine possible dose–response relationships, or emotional exhaustion effects of overexposure [11]. This research focused on immediate effects of advertising and it is unclear whether reductions in urges to drink would be sustained over time. Finally, the current findings do not indicate whether viewing alcohol warning advertising leads to immediate or long-term reductions in actual alcohol consumption.

## Conclusion

Viewing advertisements that warn about the negative consequences of alcohol use can reduce urges to drink alcohol in young adults, compared to viewing alcohol promoting or non-alcohol advertising. The mechanism of action appears to be an increase in negative affect, or displeasure, in response to the advertisements. The impact of alcohol warning advertisements upon actual consumption awaits investigation.

## Abbreviations

AUDIT, Alcohol Use Disorders Identification Test; AUQ, Alcohol Urge Questionnaire

## Additional files

Additional file 1: Supplementary material S1. Content analysis of participants’ understanding of the term ‘Drink Responsibly’. (DOCX 13 kb)

Additional file 2: Supplementary material S2. Dataset. (DAT 123 kb)
