# Supplementary material for: Viewing alcohol warning advertising reduces urges to drink in young adults: an online experiment
Source: BMC Public Health. 2016 Jul 8;16:530. doi: 10.1186/s12889-016-3192-9 (PMC4938962; doi:10.1186/s12889-016-3192-9)
Supplement: Supplementary file 1 — Supplementary material S1. Content analysis of participants’ understanding of the term ‘Drink Responsibly’. (DOCX 13 kb) [file 12889_2016_3192_MOESM1_ESM.docx]

**Supplementary material**

***Participants’ understanding of ‘Drink Responsibly’***

Participants were asked *“What do you think is meant by the term ‘Drink Responsibly’?”*, and provided an open-ended response format. A content analysis was conducted to code responses into common themes. A previous published investigation into young adults’ understanding of the term ‘drink responsibly’, which used data from university students in the United States, was used as a template for the analysis (Roznowski & Eckert, 2006).

Ten themes were identified. Table S1 presented the number and percentage of participants expressing each theme and an example response of each. Responses were coded to reflect that many participants expressed multiple themes. The theme expressed by the largest number of participants was that of limiting alcohol consumption, which included references to being aware of one’s personal limits so as not to exceed them. Only 13 participants referred to official guidance for alcohol consumption. No participants indicated that responsible drinking might mean not drinking at all.

Table S1

*Themes of participants’ understanding of the term ‘Drink Responsibly’*

| **Theme** | **Number (%) of participants expressing theme** | **Example response** |
| --- | --- | --- |
| 1. Limit consumption / awareness of personal limits | 71 (46.7%) | *“Know your limits, be aware of how much alcohol you can safely consume”* |
| 2. Maintain control and judgement over behaviour | 31 (20.4%) | *“Don't get to the stage where drinking is controlling your behaviour and causing you to act irresponsibly”* |
| 3. Health/safety of self and others | 27 (17.8%) | *“You should not drink to a degree that puts yourself or others in danger”* |
| 4. Avoid regrettable behaviour | 14 (9.2%) | *“Don't drink too much that you become aggressive, rude, or annoying”* |
| 5. Stay within recommended guidelines | 13 (8.6%) | *“To drink according to government recommendations, to avoid the negative effects of drinking too much”* |
| 6. Take personal responsibility and consider consequences of drinking | 12 (7.9%) | *“It is ok to drink, however you should always consider various consequences first”* |
| 7. Don’t get drunk | 10 (6.6%) | *“Have a drink but stop before you get drunk, socially drink”* |
| 8. Don’t binge drink (drink excessively in a short space of time) | 10 (6.6%) | *“Not drinking beyond your limits in a short-term timeframe”* |
| 9. Only drink for pleasure | 6 (3.9%) | *“Drink for enjoyment not because you need to”* |
| 10. Don’t drink and drive | 3 (2.0%) | *“Do not drive or do things you can't do when you had alcohol”* |
